# Exploiting Locality in Full Configuration Interaction Quantum Monte Carlo for Fast Excitation Generation

**DOI:** 10.1021/acs.jctc.3c00546

**Published:** 2023-12-05

**Authors:** Oskar Weser, Ali Alavi, Giovanni Li Manni

**Affiliations:** † Max-Planck-Institute for Solid State Research, Stuttgart 70569, Germany; ‡ Yusuf Hamied Department of Chemistry, University of Cambridge, Lensfield Road, Cambridge CB2 1EW, U.K.

## Abstract

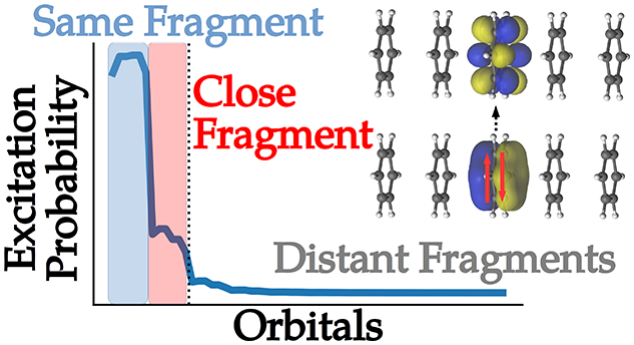

In this paper, we propose an improved excitation generation algorithm for the full configuration interaction quantum Monte Carlo method, which is particularly effective in systems described by localized orbitals. The method is an extension of the precomputed heat-bath strategy of Holmes et al., with more effective sampling of double excitations and a novel approach for nonuniform sampling of single excitations. We demonstrate the effectiveness of the algorithm for a chain of 30 hydrogen atoms with atom-localized orbitals, a stack of benzene molecules, and an Fe(II)-porphyrin model complex, whereby we show an overall efficiency gain by a factor of two to four, as measured by variance reduction per wall-clock time.

## 1 Introduction

The full configuration interaction quantum Monte Carlo (FCIQMC) method is a sparse and highly parallelizable CI eigensolver in quantum chemistry.^2,3^ Relying on the sparsity of the CI Hamiltonian and its eigensolutions, FCIQMC is in general not limited by the active space sizes,^4−12^ in contrast to conventional CI methods,^13,14^ which are limited to at most 18 electrons and 18 orbitals.^15^ Due to the nearly linear parallelization of the algorithm, FCIQMC takes full advantage of modern computer architectures^3^ and allows a large number of electrons and orbitals to be explicitly correlated; active spaces containing up to 96 electrons in 159 orbitals have been reported to date.^10^ The FCIQMC dynamics also allows to stochastically sample reduced density matrices (RDMs) via the replica trick,^16^ enabling the calculation of properties and orbital optimization, in the form of the stochastic complete and generalized active space self-consistent field (stochastic-CASSCF and stochastic-GASSCF, respectively) methods.^4,10^

In FCIQMC, the stochastically sampled wave function is represented by walkers distributed across the electronic configurations that form the CI vector space. The CI coefficient of a given configuration is proportional to the expectation value of the walker occupation on that configuration averaged over time, *c*_*i*_ ∝ ⟨*N*_*i*_^w^⟩_τ_. In the limit of large walker numbers, the approximated *c*_*i*_ values become exact. Electronic configurations corresponding to vanishingly small *c*_*i*_ coefficients are never or only rarely occupied by walkers. Since only occupied configurations have an impact on the computational costs of a FCIQMC simulation, the method relies on and takes advantage of sparsity. The initiator approximation (i-FCIQMC)^17−19^ and its further refinement, the adaptive shift method,^20,21^ increase the sparsity of the wave function and greatly improve the convergence with respect to the walker number.

The FCIQMC wave function is propagated along the imaginary time, τ, in discretized steps, Δτ. The iterative procedure of FCIQMC dynamics consists of four main stages: excitation generation, spawn, death, and annihilation. In the excitation generation stage, new configurations |*D*_*j*_⟩ are suggested as targets. In the spawn step, new walkers are then spawned to |*D*_*j*_⟩ from |*D*_*i*_⟩ (*i* ≠ *j*), with a probability *p*_spawn_ ∝ Δτ⟨*D*_*i*_|*Ĥ*|*D*_*j*_⟩. In the death step, configurations |*D*_*i*_⟩ are killed with a probability proportional to their diagonal matrix element ∝ Δτ⟨*D*_*i*_|*Ĥ*|*D*_*i*_⟩. In the annihilation step, walker contributions (arising from different spawning events) residing in the same configuration are summed with their respective signs. If they are of identical amplitude and opposite sign, they annihilate each other, and any information about that configuration is removed from the data tables.

The exact details of the four main stages of the FCIQMC algorithm greatly affect its performance. For example, a low rate of successful spawns makes the algorithm inefficient; multiple spawns from one configuration to another (a “bloom” event) cause the calculation to become unstable. In order to reduce inefficiencies, new configurations |*D*_*j*_⟩ should not be suggested uniformly in the excitation generation step. Instead, they should be selected with a probability *p*_gen_ ∝ ⟨*D*_*i*_|*Ĥ*|*D*_*j*_⟩ such that important connections are sampled more often and the spawn probability *p*_spawn_ ∝ Δτ becomes nearly constant. This nonuniform (frequently referred to as weighted) excitation generation is at the heart of an efficient FCIQMC algorithm.

So far, we used the term configurations in its full generality since the FCIQMC algorithm is not restricted to a specific basis and can use Slater determinants (SD) as well as spin eigenfunctions. In the following, we will restrict ourselves to SDs because we make use of the Slater–Condon rules in this work. In the SD basis, there exist several algorithms for the sampling of double excitations with different trade-offs between efficiency and memory demand, notable ones include: (a) the Cauchy–Schwarz excitation generator by the Alavi group,^3^ (b) the heat-bath Power–Pitzer method by Neufeld and Thom,^22^ and (c) the precomputed heat-bath (PCHB) method by Holmes et al.^1^ PCHB is the foundation of the work presented in this document.

One important property that these fast double excitation generators rely on is that the magnitude of the matrix element between two determinants, connected by a double excitation, does not depend on the involved determinants (details in Section 2). This property allows the determinant-independent precalculation of probability distributions and their efficient sampling.^1,3,22,23^ Algorithms for efficient excitation generation are also highly relevant for related methods such as selected-CI^24^ or FCI fast randomized iteration^25^ (FCI–FRI).

In PCHB, the sampling of double excitations (*AB* ← *IJ*) occurs in multiple steps. In the particle selection, a first particle *I* is chosen according to probability *p*(*I*), and then a second particle *J* is chosen under the conditional probability *p*(*J*|*I*). Next, in the hole selection, holes *A* and *B* are chosen with probabilities *p*(*A*|*IJ*) and *p*(*B*|*IJA*), respectively. When suitably localized MOs are used, after particle *I* has been selected, the second particle has a high probability, *p*(*J*|*I*), to be selected from the vicinity of *I*, rather than from spatially distant orbitals. Hence, the PCHB algorithm can take advantage of the locality effects.

Furthermore, particle indices, *I* and *J*, should preferably be guaranteed to be occupied in the starting determinant, |*D*_*i*_⟩, while the hole indices *A* and *B* should be guaranteed to be empty, which requires additional work. This implies that there is a trade-off between (a) faster sampling, which cannot guarantee occupied *I*, *J* and empty *A*, *B*, versus (b) slower but high-quality sampling, which can guarantee this. If the underlying probabilities are near-uniform, then the advantage of weighted sampling vanishes, and it might be beneficial to return back to uniform sampling, which is faster and cheaply guarantees occupied *I*, *J* and empty *A*, *B*.

The Hamiltonian matrix elements for single excitations, on the other hand, depend on the occupied orbitals and hence on the determinant from which the excitation takes place (details in Section 2). This determinant-dependency makes the precalculation of single excitation probabilities harder, and single excitations have usually been sampled uniformly.^3,10,22,1^ In order to improve upon the uniform single excitation generation, Neufeld and Thom suggested to build precalculated probabilities for single excitations relying on the reference determinant, with some modifications.^22^ This approach works best for systems featuring single-reference wave functions. In the context of variational Monte Carlo, Sabzevari and Sharma suggested contracting over all orbitals in the two-electron contribution to the single excitation Hamiltonian matrix element and to truncate all single excitations that are below a user-defined threshold.^26^ This contraction over all orbitals is independent of the determinants and can be precomputed, but the truncation introduces an artificial bias in the excitation generation; moreover, it is still uniform for all of the excitations that are above the threshold.

For delocalized molecular orbital bases, double excitations are usually more important and more numerous than single excitations. Single excitations often feature vanishingly small matrix elements, for they (partially) fulfill the Brillouin theorem in its single-reference or generalized form.^27^ Therefore, most effort in the community has gone into improving double excitation generation. However, in localized MO bases, single excitations become as (or more) important than double excitations, capturing mean field effects via the CI-expansion. The corresponding single excitation Hamiltonian matrix elements are then generally large and pivotal in wave function optimization. Thus, the development of efficient sampling schemes for single excitations becomes particularly relevant for localized orbital bases. These orbital bases play a crucial role in our recently discovered compression of spin-adapted wave functions of ground and excited states of exchange coupled polynuclear transition metal clusters.^6−8,12,28−30^

Inspired and motivated by our research carried out on localized orbital bases, we have developed a novel PCHB algorithm that, relying on the locality of the correlation effects, has an increased excitation generation efficiency for both single and double excitations. This algorithm is the focus of this work.

For our new precomputed and weighted single excitation generator, we contract over all orbitals to obtain an approximated estimate for the nonuniform sampling probabilities of single excitations. The contraction is similar to the work of Sabzevari and Sharma, but we do not introduce a bias from truncation, sample the single excitations nonuniformly, and show that the nonuniform probabilities are well approximated in localized molecular orbital bases.

For the double excitations, we systematically investigated the trade-off between speed and guaranteeing occupied or empty orbitals in PCHB and determined which combination of uniform or (constrained) weighted PCHB sampling is the fastest, to date not present in the literature. In this work, we show that the choice matters, considerably improves the excitation generation of doubles, and depends on the system at hand. We also improve upon the current literature by examining how to efficiently draw from constrained distributions, for example *p*(*I*)|_*I*∈|*D*_*i*_⟩_ from the precomputed *p*(*I*).

The new PCHB algorithm for singles and the improved excitation generation for doubles allows FCIQMC dynamics that are two to four times more efficient than the implementations developed by Holmes et al.^1^ and by us (within the NECI^3^ project).

The novel PCHB algorithm is easily extended to the recently developed stochastic-GAS wave function optimizations^10^ and to the spin-purification strategy in SD based FCIQMC.^11^

The remainder of the article is organized as follows: in Section 2, we give the necessary background for our novel PCHB excitation generator in FCIQMC. This includes the equations for the evaluation of Hamiltonian matrix elements, their decay with increasing spatial distance, and the already existing PCHB algorithm for double excitations; in Section 3, we introduce our improved sampling of double excitations; in Section 4, we introduce our new weighted, precomputed single excitation generation; in Section 5, we demonstrate the application of the novel PCHB on several test case molecular systems and quantify the efficiency improvements: a chain of 30 hydrogen atoms with atom-localized orbitals, whose Hamiltonian is dominated by single excitation couplings, demonstrates the capabilities of our new algorithm for sampling single excitations; stacks of benzene molecules, with fragment-localized MOs serves as an example for a system characterized by strong correlation effects (double excitations) within each fragment, and generally small correlation effects across the fragments; the efficiency of the new PCHB is numerically demonstrated also for highly delocalized MOs, using an Fe-porphyrin model system from previous works,^4,5,9,10^ as well as the N_2_ dimer at equilibrium geometry with a large full-CI space. In Sections 6 and 7, we discuss how both generalized active spaces and spin purification on an SD basis benefit from the improved PCHB algorithm. Section 8 collects our final remarks and conclusions from the work.

## 2 Theoretical Background

This section describes the key elements that represent the foundation of the novel PCHB algorithm suggested in the present work.

### 2.1 Matrix Elements

Using the conventions of the “purple book”^27^ and assuming orthonormal spin–orbitals, we write the second-quantized, nonrelativistic molecular Hamiltonian operator in the Born–Oppenheimer approximation with atomic units as
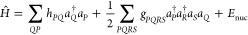
1where *P*, *Q*, *R*, and *S* are generic spin–orbital indices. The *a*^†^ and *a* are the usual unitary, second-quantized creation and annihilation operators. The one-electron integral *h*_*AI*_ is given as
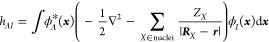
2where ϕ_*I*_ denotes the *I*-th spin–orbital, ***r*** and ***x*** refer to the spatial and the combined spatial and spin electronic coordinate, respectively, and *Z*_*X*_ and ***R***_*X*_ are the charge and spatial coordinate of the *X*-th nucleus. The two-electron integral *g*_*AIBJ*_ is given in chemist’s notation as
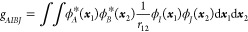
3where *r*_12_ = |***r***_1_ – ***r***_2_|. The nuclear repulsion term, *E*_nuc_, is the classical repulsion of point charges given by
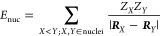
4

The Hamiltonian operator contains up to two-particle excitation terms and in general, the matrix element ⟨*D*_*i*_|*Ĥ*|*D*_*j*_⟩ between two determinants is zero if they differ by occupation in more than two spin–orbitals (in virtue of the Slater–Condon rules^31^). The nonzero terms are given as
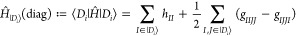
5
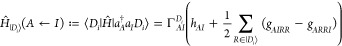
6
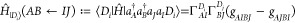
7where indices *I* < *J* and *A* < *B* refer to occupied and unoccupied spin–orbitals in |*D*_*i*_⟩, respectively, connecting to |*D*_*j*_⟩. We write *I* ∈ |*D*_*i*_⟩ if *I* is occupied in determinant |*D*_*i*_⟩; hence, *R* in eq 6 represents an index running over all the occupied spin–orbitals. The Fermionic prefactor is Γ_*AI*_^*D*_*i*_^ = (−1)^*n*^, where *n* is the number of occupied spin–orbitals between *A* and *I* in determinant |*D*_*i*_⟩. Note also that the indices for the single and double excitations have to be pairwise different (*I* ≠ *J*, *A* ≠ *B*, {*I*, *J*}∩{*A*, *B*} = ⌀). We will write uppercase *I* for spin–orbitals, lowercase *i* for spatial orbitals, and *i*_σ_ for the *i*-th spatial orbital with spin σ.

Our new PCHB excitation generator relies on the decay of Hamiltonian matrix elements from vanishing integrals. We assume the MOs to be suitably localized and separated from each other so that a distance *R* between the MOs can be defined. We have to emphasize that the following discussion does not apply to delocalized orbitals. For large enough distances, the one-electron integrals decay with (*S*_*IA*_), where *S*_*IA*_ = ⟨|ϕ_*I*_|||ϕ_*A*_|⟩ is the absolute overlap between the two spin–orbitals, which decays as (exp(−*R*_*AI*_^2^)) with the distance *R*_*AI*_ between the MOs ϕ_*A*_ and ϕ_*I*_.^32^ An intuitive understanding comes from the observation that with less and less overlap, the two functions ϕ_*I*_ and ϕ_*A*_ have more and more disjoint support with respect to the integration variable (see eq 2).

For the decay of the two-electron integrals *g*_*AIBJ*_, we need to define the following quantities: *S*_*IA*_ and *R*_*IA*_ are defined as before, *S*_*JB*_ and *R*_*BJ*_ are the absolute overlaps and distances between ϕ_*J*_ and ϕ_*B*_, and *R*_ovl,*AI*,*BJ*_ is the distance between the overlap distributions ϕ_*A*_ϕ_*I*_ and ϕ_*B*_ϕ_*J*_. With this notation, the two-electron integrals *g*_*AIBJ*_ decay for large distances as .^33^ We now look at specific, important integrals. In the case of the classical Coulomb term *g*_*IIJJ*_, we have *S*_*II*_ = *S*_*JJ*_ = 1, which implies a decay of *g*_*IIJJ*_ with  (note *R*_ovl,*II*,*JJ*_ = *R*_*IJ*_ in this case). This decay can be related to classical electrostatics, since the orbitals depending on the same electronic coordinate can be combined into the classical electron density ρ_*I*_ = ϕ_I_*ϕ_*I*_ and decays, like classical point charges, with  for a large-enough distance between the MOs ϕ_*I*_ and ϕ_*J*_. Now we look at specific examples of nonclassical two-electron integrals that appear in the single-excitation matrix elements, namely, *g*_*AIRR*_ or *g*_*ARRI*_. The integral *g*_*AIRR*_ decays with *S*_*AI*_ and hence exponentially with *R*_*AI*_. The exchanged version *g*_*ARRI*_ decays with . Due to the triangle inequality in Euclidean space, we can write *R*_*AR*_ + *R*_*RI*_ ≥ *R*_*AI*_ and *R*_ovl,*AR*,*RI*_ ≥ *R*_*AI*_, which then gives an overall exponential decay of the integral *g*_*ARRI*_ with *R*_*AI*_. As shown in eq 6, integrals *g*_*AIRR*_ or *g*_*ARRI*_ are the only ones appearing in the single excitation matrix elements; thus, we expect an exponential decay with *R*_*AI*_. This observation is crucial for the weighted singles excitation generator.

### 2.2 Excitation Probabilities

The aim of excitation generators in FCIQMC is to suggest a new determinant, |*D*_*j*_⟩, from |*D*_*i*_⟩, with a probability proportional to their matrix element. So the optimal excitation generator would sample with the following probabilities
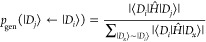
8Here and in the following, we write |*D*_*i*_⟩ ∼ |*D*_*j*_⟩ if two determinants are connected, and |*D*_*j*_⟩ ← |*D*_*i*_⟩ for a specific excitation from |*D*_*i*_⟩ to |*D*_*j*_⟩. Moreover, the labels |*D*_*i*_⟩|*D*_*j*_⟩ or |*D*_*i*_⟩|*D*_*j*_⟩ are used to indicate whether the two determinants are connected via single or double excitations, and |*D*_*j*_⟩|*D*_*i*_⟩ or |*D*_*j*_⟩|*D*_*i*_⟩ to indicate whether |*D*_*j*_⟩ is obtained via single or double excitations from |*D*_*i*_⟩ to |*D*_*j*_⟩.

We first decide with probability *p*_1_ or *p*_2_ whether to perform a single or double excitation, respectively, and then sample from the conditional probabilities *p*_gen_(|*D*_*j*_⟩ ← |*D*_*i*_⟩|*n*), *n* ∈ {1, 2}. Here and in the following, subscripts 1 and 2 refer to single and double excitations, respectively, while superscripts refer to the algorithm, e.g., *p*_2_^PCHB^ is a PCHB-weighted double excitation probability. These subscripts or superscripts are omitted if the expressions are not restricted to a specific algorithm or level of excitation.

During a calculation, the time step is optimized to be as high as possible while preserving a stable calculation. In the simplest approach, this is achieved by
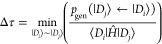
9which keeps the spawn probability Δτ below unity for all previously visited determinant pairs |*D*_*i*_⟩ ∼ |*D*_*j*_⟩.

In order to maximize the possible time step Δτ from eq 9, the probabilities *p*_1_ and *p*_2_ are adjusted during the calculation to preserve
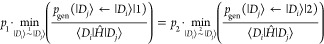
10From eq 10, it becomes apparent that if an excitation generator is of low quality, i.e., there is a pair |*D*_*i*_⟩  |*D*_*j*_⟩ with a low ratio  then the corresponding *p*_*n*_ become large. Note that there exist more elaborate schemes to update the time step, for example, the histogramming Δτ search, which does not depend only on the worst case unlike eq 9.^34^ Even in such schemes, the overall trend is retained; i.e., if single excitations are suboptimally sampled, they will be sampled more often.

Localized MOs tend to have considerably large matrix elements for single excitations, and these matrix elements are nonuniform, because excitations to distant MOs are negligible (vide infra). The generally utilized uniform excitation generation for single excitations, in the case of localized orbital bases, would lead to small *p*_gen_ for single excitations due to the numerous vanishingly small single connections among distant MOs, leading to exaggeratedly large *p*_1_ values, and overshadow any improvement of PCHB for double excitations. In these cases, the uniform excitation generator for single excitations represents a particularly poor choice and would negatively impact the entire dynamics. The present work overcomes such limitations and provides a more balanced excitation generation.

### 2.3 Original PCHB Strategy

In this section, we summarize the key elements of the original work on PCHB by Holmes and co-workers.^1^ The exact weighted sampling of orbitals for double excitations from |*D*_*i*_⟩ is given by
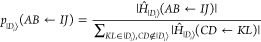
11

Clearly, whether the element is zero or nonzero depends on occupation (*I*, *J* ∈ |*D*_*i*_⟩ and *A*, *B* ∉ |*D*_*i*_⟩), and hence on the determinant. When nonzero, double excitations have the important property that the magnitude is independent of the starting determinant |*D*_*i*_⟩ (compare eq 7). If we write for the weight
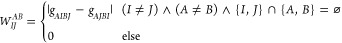
12then eq 11 can be factorized without approximations, as
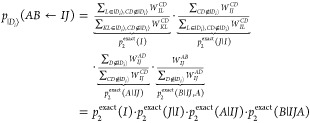
13

The crucial approximation of the original PCHB is the contraction over all indices and hence the determinant-independent precalculation of probabilities, which are given by
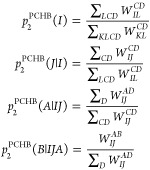
14

Considering that the order in which we pick the particles and holes does not matter for the final determinant, and that in general, one cannot assume symmetry, e.g., *p*(*I*|*J*) ≠ *p*(*J*|*I*), the full probability of selecting |*D*_*j*_⟩ = *a*_*A*_^†^*a*_*B*_^†^*a*_*J*_*a*_*I*_|*D*_*i*_⟩ can then be calculated via
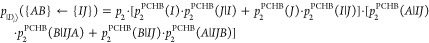
15

The precomputed probabilities from eq 14 have the huge advantage that they can be calculated once at the beginning of the calculation. In combination with the alias sampling algorithm,^23,35^ the precalculated probabilities allow sampling with (1) run-time cost. This is a typical trade-off between memory and time. The memory requirements can in part be reduced by using read-only, node-shared memory for storing the alias tables.^3,10^

It would be desirable for an excitation generator that particle indices, *I*, *J*, and hole indices, *A*, *B*, are guaranteed to be occupied and unoccupied in |*D*_*i*_⟩, respectively. Unfortunately, sampling according to eq 14, with no reference to the current determinant, |*D*_*i*_⟩, has the important disadvantage that the particle/hole indices suggested might not be occupied/empty in |*D*_*i*_⟩; this excitation can be discarded.

## 3 Improved PCHB for Double Excitations

Our improvements to the sampling of doubles in PCHB consists of determining when and how to guarantee if orbitals are occupied or empty. The necessary renormalizations for constrained sampling (a sampling where the notion of occupied/empty orbital is available) that guarantees *I*, *J* and *A*, *B* to be occupied and empty, respectively, in |*D*_*i*_⟩, read as
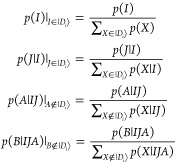
16

The cost to evaluate these probabilities scales with the number of terms in the sum as (*N*_e_) and (*n*_orb_ − *N*_e_) for *I*, *J* and *A*, *B*, respectively.

A clear trade-off emerges from the comparison of eqs 14 and 16, between (a) drawing from precomputed distributions with constant scaling and subsequently discarded excitations, and (b) from ad hoc constrained probability distributions with linear scaling and no discarded excitations. We will refer to unconstrained sampling (*p*(*I*), ···) as *fast-weighted* (eq 14) and to constrained sampling (*p*(*I*)|_*I*∈|*D*_*i*_⟩_,_···_) as *fully weighted* sampling (eq 16). The *uniform* sampling, also used for comparison in this work, allows drawing from constrained subsets with (1) time. This implies that for near-uniform probability distributions, the uniform sampling might outperform weighted sampling after taking the quality of excitation and iteration time into account. The possible sampling schemes are summarized in Table 1 and, in principle, can be freely combined among each other for the selection of first/second particle or hole. Note that fast-weighted and fully weighted methods have additional, non-negligible memory and one-time initialization cost, that scale with (*n*_orb_^1^), (*n*_orb_^2^), (*n*_orb_^3^), and (*n*_orb_^4^) for the first and second particles and first and second holes, respectively. We will see later that the specific choice of combinations has large implications for the performance of the overall FCIQMC dynamics. In particular, fast-weighted sampling works best for the hole-selection of active spaces with an excess of unoccupied orbitals, i.e., if there are many empty orbitals, then *p*_2_^PCHB^ (*A*|*IJ*) ≈ *p*_2_^PCHB^ (*A*|*IJ*)|_*A*∉|*D*_*i*_⟩_, and performing the full weighting would only result in additional, unnecessary computational effort. Similar conclusions would be reached for active spaces with an excess of occupied orbitals, but those are more rare in practical applications and limited by the Pauli principle and will not be discussed any further. Uniform sampling works best for active spaces with near-uniform excitation probabilities and has no additional costs to guarantee orbitals to be occupied or unoccupied. Fully weighted sampling is the slowest in wall-clock time but samples with the highest quality.

**Table 1:** Different Sampling Methods and Their Run-Time Scaling

method	distribution	run time scaling
fast-weighted	*p*_2_^PCHB^(*I*), *p*_2_^PCHB^(*J*|*I*), *p*_2_^PCHB^(*A*|*IJ*), *p*_2_^PCHB^(*B*|*IJA*)	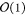
fully weighted	,	or
uniform	,	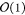

To date, no systematic investigation is available on which combination of uniform or (constrained) weighted PCHB sampling is the fastest. The original PCHB paper^1^ and the work by Neufeld and Thom^22^ assumed fully weighted particle selection (*p*_2_^PCHB^(*I*)|_*I*∈|*D*_*i*_⟩_ and *p*_2_^PCHB^(*J*|*I*)|_*J*∈|*D*_*i*_⟩_) and fast-weighted hole selection (*p*_2_^PCHB^(*A*|*IJ*) and *p*_2_^PCHB^(*B*|*IJA*)) to be the fastest combination. In our earlier work using PCHB,^3,10^ we have assumed uniform particle selection (*p*_2_^uni^(*IJ*)|_*I*,*J*∈|*D*_*i*_⟩_) and fast-weighted hole selection (*p*_2_^PCHB^(*AB*|*IJ*)) to be the fastest combination. Note that directly sampling pairs *IJ* and *AB* can reduce the memory cost, as described in ref (3), but is tied to a fast-weighted sampling scheme.

Based on the previous discussion, our improvements to the sampling of double excitations in PCHB are two-fold: (a) we suggest optimal combinations of fast, fully weighted, and uniform sampling depending on general features of the systems investigated, and (b) we improve the sampling speed from constrained distributions. We present these improvements separately in the next two subsections.

### 3.1 Improved Sampling Combinations

The choice of better combinations of sampling schemes greatly depends on the probability distributions from eq 14 which are highly system dependent. Different systems may prefer different excitation generation schemes based on the form of electronic interactions, the orbital basis of choice (delocalized or localized), and whether there is an excess of empty orbitals. We will analyze the shape of probability distributions for some selected systems, and numerically prove our findings in the application Section 5.

The first example we consider is a stack of ten benzene molecules at an intermolecular distance of 3.0 Å, and orbitals that are fragment-localized, i.e., they are natural, Hückel-like orbitals on each benzene. The CAS (60,60) was ten times the minimal active space of six electrons in six π-orbitals for one benzene unit. As we showed in a previous publication,^10^ the system as a whole is dominated by excitations inside each fragment. Additionally, incorporating single excitations between the fragments is enough to recover the full CI energy.

**Figure 1:**
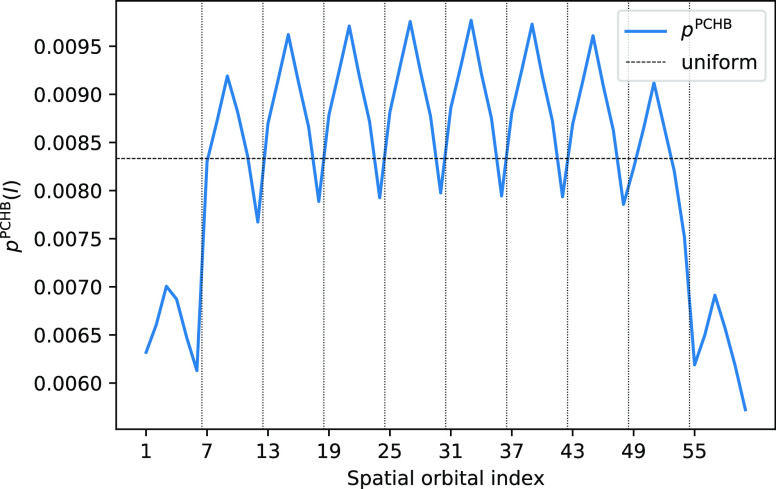
PCHB probability of the first particle *p*_2_^PCHB^(*I*) for a double excitation in a stack of 10 benzene molecules. The orbitals are ordered by fragment, and the vertical dotted lines separate MOs of different benzene molecules. Since α and β electrons have the same probability, assuming RHF-type orbitals, we could use the spatial orbital index. The horizontal dashed line gives the probability of the corresponding unconstrained uniform distribution *p*_2_^uni^(*I*) as reference. The value is obtained as 1/*n*_orb_ = 1/120 = 8.3 × 10^–3^.

The probability distribution *p*_2_^PCHB^(*I*) is displayed in Figure 1. The symmetry of repeating fragments is visible in the probabilities, and the fragments at the border have slightly different behavior than the fragments in “bulk”. However, overall, it is a near-uniform distribution. For this reason, *p*_2_^uni^(*I*)|_*I*∈|*D*_*i*_⟩_ is likely the best choice for the first particle, cheaply guaranteeing *I* to be occupied. This section is about qualitative trends and gives robust rules of thumb for selecting the sampling methods. It is still necessary to measure timings with the system at hand, as can be seen in the numerical results of Section 5.

**Figure 2:**
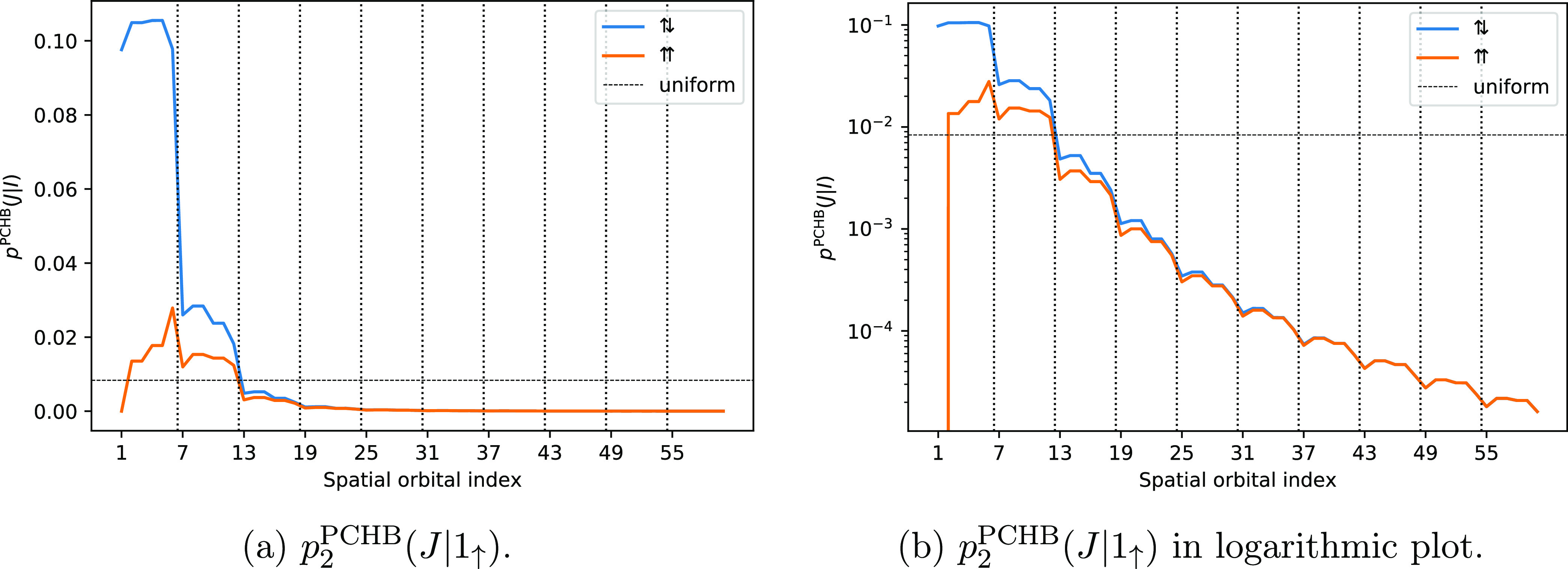
PCHB probability of the second particle after having picked an α electron in the first spatial orbital *p*_2_^PCHB^(*J*|1_↑_) for a double excitation in a stack of 10 benzene molecules. The orbitals are sorted by fragment, and the vertical dotted lines separate MOs of different benzene molecules. We again use spatial orbital indices. The blue line represents an opposite spin *p*_2_^PCHB^(*j*_↓_|1_↑_), while the orange line represents a parallel spin *p*_2_^PCHB^(*j*_↑_|1_↑_) for the second electron. The right figure is a logarithmic version of the left one. The horizontal dashed line gives the probability of the corresponding unconstrained uniform distribution *p*_2_^uni^(*J*|1_↑_) as the reference. The value is obtained as 1/*n*_orb_ = 1/120 = 8.3 × 10^–3^.

The conditional probability of *p*_2_^PCHB^(*J*|*I*) to sample the second particle is displayed in Figure 2. The left, nonlogarithmic plot (Figure 2a) shows that the correlation is highly local. The second particle has the highest probability of being selected on the same fragment, while quickly decaying for neighboring fragments. Any choice for the second particle further away than 6 Å (second-nearest fragment) is nearly negligible. The logarithmic plot (Figure 2b) reveals the exponential decay with increasing distance. This observation shows that the distribution is highly nonuniform and weighted sampling is preferred. Moreover, as the orbital space considered, CAS (60,60) is far from a case with an excess of occupied orbitals; it is advantageous to guarantee that the second picked orbital index is indeed occupied. By this argument, fully weighted sampling with *p*_2_^PCHB^(*J*|*I*)|_*J*∈|*D*_*i*_⟩_ is recommended. In addition, the weighted sampling automatically prefers geminal, i.e., opposite spin pair, excitations, as can be seen from comparing the blue and orange lines in Figure 2.

The subsequent hole-selections are also highly nonuniform with no excess of empty orbitals, which suggests
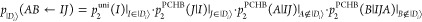
17to be the best choice for such localized systems, namely, uniform selection for the first particle and fully weighted selection for the second particle and the two holes. Notably, this sampling scheme is neither the one suggested by Holmes et al.^1^ nor the one we have been using in NECI.^3^

The second system is full CI on the N_2_ dimer at equilibrium distance with a Dunning’s cc-pVQZ basis^36^ using Hartree–Fock orbitals, resulting in a (14,110) active space. This is a single-reference system with highly delocalized orbitals that fulfills the Brillouin theorem, which makes the double excitations particularly important.

**Figure 3:**
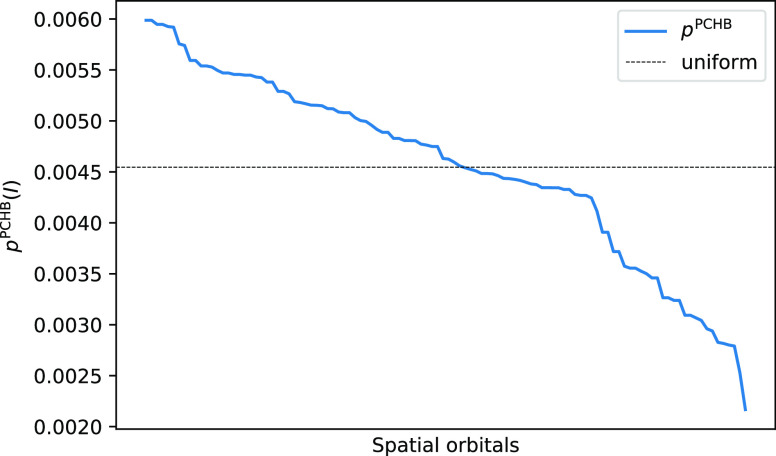
PCHB probability of the first particle *p*_2_^PCHB^(*I*) for a double excitation in the N_2_ dimer. The orbitals are sorted by probability. Since α and β electrons have the same probability, assuming RHF-type orbitals, we could use the spatial orbital index. The horizontal dashed line gives the probability of the corresponding unconstrained uniform distribution *p*_2_^uni^(*I*) as reference. The value is obtained as 1/*n*_orb_ = 1/220 = 4.5 × 10^–3^.

The probability for the first particle, *p*_2_^PCHB^(*I*), is displayed in Figure 3 and has a small variation. For this reason, analogously to the benzene stack, *p*_2_^uni^(*I*)|_*I*∈|*D*_*i*_⟩_ is considered to be the best choice for the first particle.

**Figure 4:**
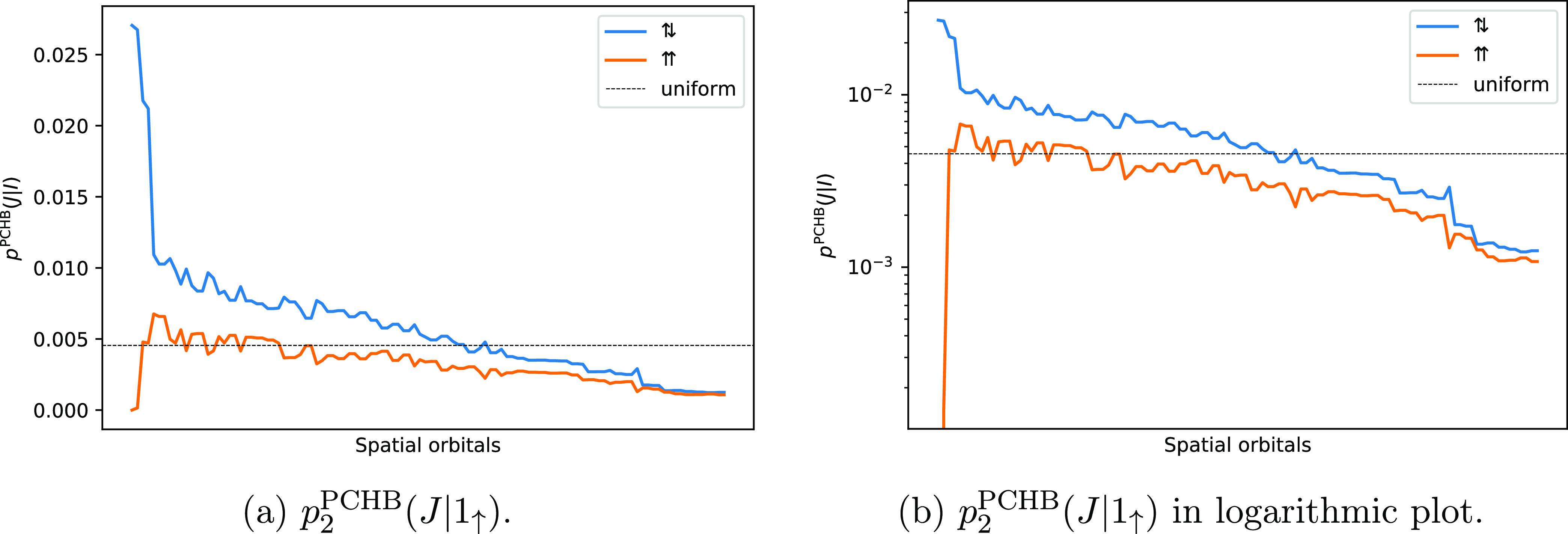
PCHB probability of the second particle after having picked an α electron in the first spatial orbital *p*_2_^PCHB^(*J*|1_↑_) for double excitation in the N_2_ dimer. We use again spatial orbital indices, which are sorted by joint spatial probability, i.e., *p*_2_^PCHB^(*j*_α_|1_↑_) + *p*_2_^PCHB^(*j*_β_|1_↑_). The blue line represents an opposite spin *p*_2_^PCHB^(*j*_↓_|1_↑_), while the orange line represents a parallel spin *p*_2_^PCHB^(*j*_↑_|1_↑_) for the second electron. The right figure is a logarithmic version of the left one. The horizontal dashed line gives the probability of the corresponding unconstrained uniform distribution *p*_2_^uni^(*J*|1_↑_) as reference. The value is obtained as 1/*n*_orb_ = 1/220 = 4.5 × 10^–3^.

The conditional probability *p*_2_^PCHB^(*J*|*I*) to sample the second particle is displayed in Figure 4. The left, nonlogarithmic plot (Figure 4a) is nonuniform; however, the range of PCHB values is much smaller than for the stack of benzene (Figure 2a), indicating a more homogeneous distribution. The logarithmic plot (Figure 4b) reveals that the probabilities range mostly over “just 1” order of magnitude, unlike the 4 orders of magnitude in Figure 2b. There is no obvious exponential decay for the probability of the second particle due to the compact nature of the system. Overall, the N_2_ dimer and similar systems might still benefit from uniform sampling of the second particle *p*_2_^uni^(*J*|*I*)|_*J*∈|*D*_*i*_⟩_. This suggestion is investigated in greater detail and confirmed in Section 5.

The subsequent hole-selections are again highly nonuniform, which suggests weighted sampling. Due to the excess of empty orbitals, the fast-weighted sampling of hole indices is expected to outperform the fully weighted strategy since a randomly picked index is more likely to be unoccupied. The above considerations indicate the following sampling scheme
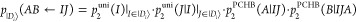
18for N_2_ and similar compact systems featuring delocalized orbital bases and active spaces with an excess of empty orbitals. Notably, this sampling scheme is the choice we have been using in NECI^3^ and differs from the one suggested by Holmes et al.^1^

### 3.2 Faster Constrained Sampling

This subsection describes how to efficiently draw from constrained distributions, for example, *p*_2_^uni^(*I*)|_*I*∈|*D*_*i*_⟩_ from the precomputed *p*(*I*). We assume that both the unconstrained probabilities *p*(*I*) and the corresponding alias tables for alias sampling are preconstructed, i.e., we can draw in (1) from *p*(*I*).

In the literature, it has so far been assumed that constrained distributions are best sampled by rebuilding alias tables for the constrained probabilities *p*(*I*)|_*I*∈|*D*_*i*_⟩_.^22,1^ This approach still uses the precomputed probabilities *p*(*I*) to obtain *p*(*I*)|_*I*∈|*D*_*i*_⟩_ but ignores the already available alias tables for *p*(*I*). This is an unnecessary slowdown because the computational effort for reconstructing constrained alias tables scales linearly with the number of particles  or the number of empty orbitals  for constraints of the type *I* ∈ |*D*_*i*_⟩ or *A* ∉ |*D*_*i*_⟩, respectively.

We suggest to use rejection sampling, where *p*(*I*) takes the role of the candidate density and the constrained *p*(*I*)|_*I*∈|*D*_*i*_⟩_ takes the role of the target density.^38^ This means we redraw from *p*(*I*), using the already available alias tables, until the required (un)occupied index is sampled (see alg. 3). If |*D*_*i*_⟩ is represented as a bit mask, then the test for membership *I* ∈ |*D*_*i*_⟩ has  cost, and since drawing from *p*(*I*) also has  cost, the whole operation of redrawing has  scaling with respect to system size.

Note that the constant scaling with respect to the system size is only one part of the story. If the weight of the subset becomes low, then the probability of misses becomes high and the expected number of samples is given by . For this reason, it can be advantageous to avoid rejection sampling, if the renormalization constant ∑_*I*∈|*D*_*i*_⟩_*p*(*I*) is small (see algorithm-line 3.10). It also means that rejection sampling of holes works better for systems with a large number of empty orbitals, while rejection sampling of particles works better for systems with a small number of empty orbitals.

In order to obtain the renormalized probabilities (eq 16), we need equations of the type *p*(*I*)|_*I*∈|*D*_*i*_⟩_ = *p*(*I*)/(∑_*X*∈|*D*_*i*_⟩_*p*(*X*)), which would scale with  because of the sum at the denominator; for hole constraints *p*(*A*|*I*|_*A*∉|*D*_*i*_⟩_ = *p*(*A*|*I*)/(∑_*X*∈|*D*_*i*_⟩_*p*(*X*|*I*))the scaling would be . Since there is the following relationship for the complement
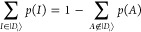
19we can choose to scale with the number of particles or empty orbitals, whichever is cheaper. This is particularly advantageous for active spaces with an excess of empty orbitals.

**Figure 5:**
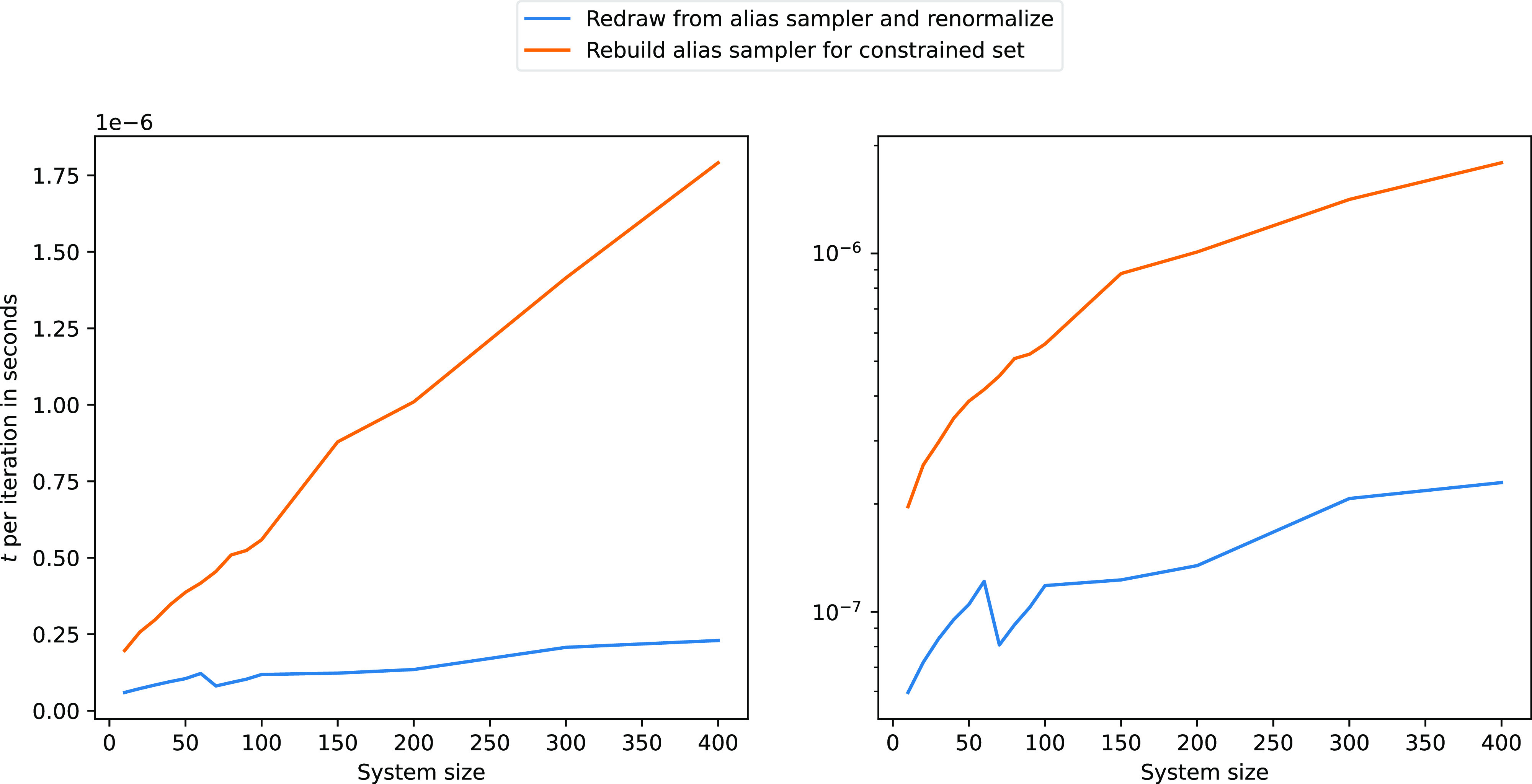
Sampling time for drawing from a constrained subset that contains half of the elements of the total system size using two different algorithms: redrawing method (blue line, algorithm 3) and rebuilding an alias table for the constrained subset (orange line). The right figure is a logarithmic version of the left one.

Figure 5 depicts the typical situation of an active space with a similar number of particles and spatial orbitals; hence, the constrained subset, for both particle- and hole-selection, contains half of the elements of the total system size. In the same figure, we can see the linear scaling for both methods and that the redrawing method is roughly 5 times faster than rebuilding the alias sampling tables. For higher weights of the constrained subset, the redrawing method can get up to 10 times faster. Further details of the timings are discussed in the Appendix A.1.

In the context of FCIQMC, the faster sampling from constrained distributions is relevant for both single and double excitations; the singles are described in the next section.

## 4 Novel Scheme for Precomputed Single Excitations

As discussed earlier, an accurate and balanced generation of single and double excitations is of paramount importance for efficient FCIQMC dynamics. According to eq 10, a suboptimal single excitation generator leads to an exceedingly frequent sampling of single excitations, which overshadows any improvement for double excitation generators.

In this section, we introduce an efficient way to precompute the probabilities for single excitations. As for the precomputed double excitations, we start with the exact, determinant-dependent, factorized sampling probability
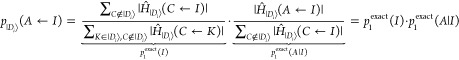
20

Equation 20 depends in two ways on the starting determinant |*D*_*i*_⟩. The first, obvious dependence, is the restricted summation over (un)occupied orbitals (the *K* and *C* indices). This dependence is also present for double excitations and is approximated in PCHB by contracting over all orbitals (eq 13). The additional dependence for single excitations is that the magnitude of the matrix element highly depends on the starting determinant (see eq 6). For example, according to the Brillouin theorem, single excitations from the Hartree–Fock reference determinant |*D*_HF_⟩ should have a vanishing matrix element
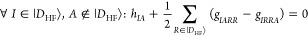
21even though the individual integrals might have a high magnitude. The same *A* ← *I* excitation from an excited determinant can have a large nonzero matrix element just because of the contraction over different two-electron integrals. This is at the core of why it is hard to approximate and precompute determinant-independent single-excitation sampling probabilities.

In the special case of localized MO bases, and under the assumption that *I* and *A* are from spatially distant orbitals, we have |*h*_*IA*_| ≈ |*g*_*IARR*_| ≈ |*g*_*IRRA*_| ≈ 0, i.e., the single excitations become vanishingly small regardless of the determinant. In light of this property, we suggest the approximation of contracting over all orbitals (*R*) for the weight *S*_*I*_^*A*^
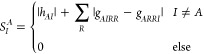
22

Due to the triangle inequality (|*A* + *B*| ≤ |*A*| + |*B*|), it follows that for all determinants, |*Ĥ*_|*D*_*i*_⟩_(*A* ← *I*)| ≤ *S*_*I*_^*A*^. Next, as for the doubles (see eq 14), we contract over all orbitals, thus further approximating the exact expression for single excitations (eq 20), to yield
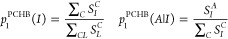
23

In Table 2, we list all possible combinations of probability distributions for single excitations. As in the case of doubles (Table 1), we can draw from constrained subsets and guarantee the sampled indices to be occupied or unoccupied. Sometimes, it might be even beneficial to return back to uniform sampling if the probability distribution is uniform enough.

**Table 2:** Different Sampling Methods for Single Excitations and Their Scaling

method	distribution	scaling
fast-weighted	*p*_1_^PCHB^(*I*), *p*_1_^PCHB^(*A*|*I*)	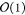
fully weighted	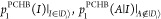	or
uniform	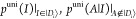	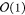

**Figure 6:**
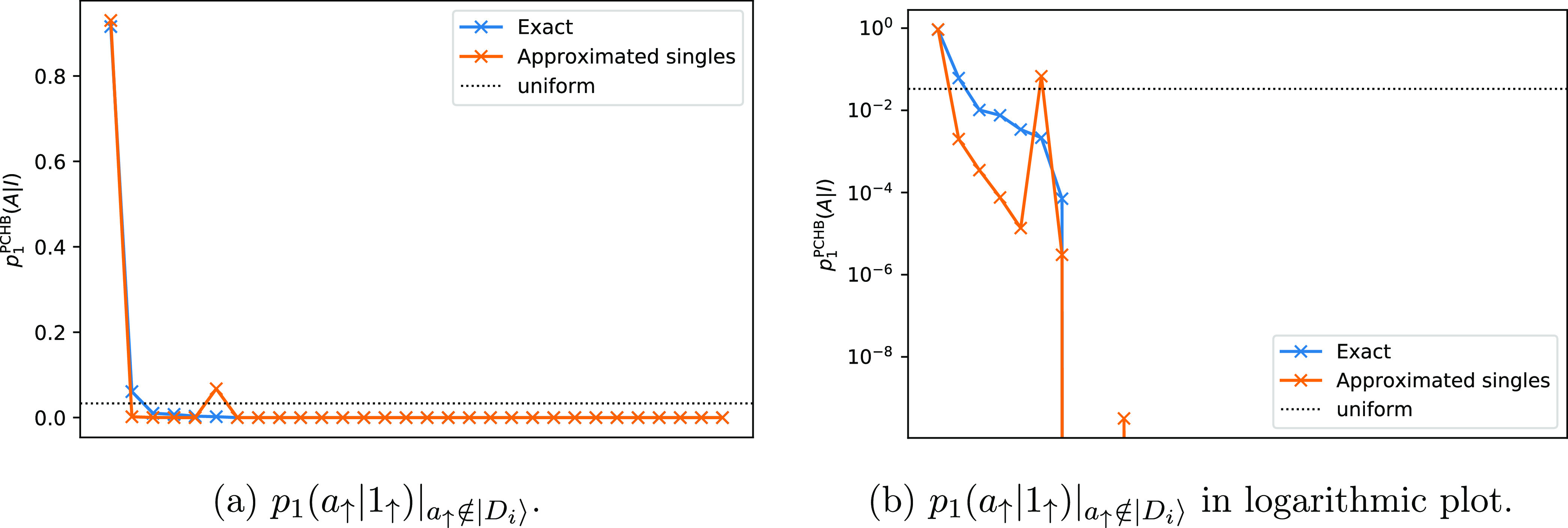
PCHB probability of the hole in a single excitation after having picked an α electron in the first spatial orbital, *p*_1_^PCHB^(*a*_↑_|1_↑_), on a stack of 10 benzene molecules. The blue line shows the exact probability (eq 20) assuming that the starting determinant |*D*_*i*_⟩ = |2, 2, 2, 0, 0, 0; 2, ···⟩ is given by doubly occupying all π-orbitals and keeping all π*-orbitals empty for each benzene molecule. The orange line shows the approximated PCHB probabilities for single excitations (eq 23). Only unoccupied α spin–orbitals, i.e., allowed holes, are considered. The orbital indices are sorted by decreasing exact probability (blue line). (b) Logarithmic scale of (a). The horizontal dashed line gives the probability of the corresponding unconstrained uniform distribution *p*_1_^uni^(*a*_↑_|1_↑_)|_*a*↑∉|*D*_*i*_⟩_ as reference. The value is obtained from the inverse of the number of empty α-orbitals 1/(*n*_orb_^↑^ – *N*_e_^↑^) = 1/30 = 3.3 × 10^–2^.

We have investigated whether eq 23 approximates the exact probabilities well and which of the combinations from Table 2 is the most efficient sampling scheme for single excitations. In Figure 6, the *p*_1_^PCHB^(*a*_↑_|1_↑_)|_*a*↑∉|*D*_*i*_⟩_ values are shown for the stack of 10 benzene molecules with fragment-localized MOs (the probability of drawing the hole *a*_↑_ after having drawn a ↑-particle from the first spatial orbital). For the exact probability (eq 20), we assume that we start from the Hartree–Fock reference determinant, which is given by doubly occupying all π-orbitals and keeping all π*-orbitals empty (blue line in Figure 6) The orange line shows the corresponding approximated probability *p*_1_^PCHB^(*a*_↑_|1_↑_)|_*a*↑∉|*D*_*i*_⟩_ (eq 23). Figure 6 shows good agreement between the approximated and exact single excitations. It also confirms the exponential decay of matrix elements with the distance and the importance of excluding spatially distant orbitals, which would be unnecessarily sampled in a uniform scheme.

The probability for selecting the particle *p*_1_^PCHB^(*I*)|_I∈|*D*_*i*_⟩_ is near-uniformly distributed. As for the double excitation case, the particle, *I*, can safely be sampled uniformly, and
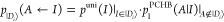
24is predicted to be the fastest sampling scheme for single excitations.

Apart from the choice of the combination of sampling schemes, we also mention that the singles benefit from the improved sampling from constrained distributions, as discussed in Subsection 3.2.

As a final remark, we would like to emphasize that the new excitation generation for singles and the improved sampling combination of doubles can also be applied to methods such as selected-CI^24^ or FCI-FRI.^25^ When combined with those methods, localized orbital bases mainly benefit from the new weighting scheme for single excitations, and the ratio of particles to empty orbitals determines whether fully weighted or fast-weighted particle or hole-selection is to be preferred. However, some differences between FCIQMC and FCI-FRI are to be expected; for example, the number of empty orbitals where fully weighted hole-selection becomes better than fast-weighted for a given system might differ for the two approaches. Furthermore, in FCI-FRI, additional techniques can be applied to obviate the need for renormalization.^25^

## 5 Applications

Four test case applications are discussed in the following to numerically show the improved performance of the novel excitation generators: (a) a chain of 30 hydrogen atoms with atom-localized orbitals, therefore dominated by single excitations, demonstrates the capabilities of our new algorithm for sampling single excitations, (b) a stack of different numbers of benzene molecules with fragment-localized MOs serves as an example for a system of fragments with highly localized correlation effects that is dominated by double excitations and highlights the improved sampling of double excitations, (c) an Fe-porphyrin model complex shows the efficiency of the new single excitation sampling for systems with mostly delocalized MOs, as long as there is some spatial separation, and (d) the N_2_-dimer with Hartree–Fock orbitals shows how a uniform particle selection for the double excitations can improve the performance of PCHB.

### 5.1 Computational Details

Orbital optimization (e.g., the Hartree–Fock method), their transformations (e.g., localization), integral evaluations, their transformation to the basis of active molecular orbitals, and their storage on the formatted FCIDUMP(39) file were performed using OpenMolcas.^40,41^ The new excitation generation has been implemented in a locally modified version of NECI(3) and will be made publicly available via our open-source repository^42^ upon publication of this work. The different sampling schemes are compared by their variance reduction per wall-clock time . FCIQMC dynamics were entirely run in stochastic mode, i.e., no semistochastic propagation was enabled. For every system and every algorithm, there were three calculations of 24 h; the efficiency was then averaged over these three runs. Since the variance of the projected energy has to be estimated under stationary conditions, all calculations started from walker population distributions of already converged wave functions.^43^ To guarantee the optimal Δτ, *p*_1_, and *p*_2_ for each algorithm, we optimized these values for 5 × 10^4^ iterations according to eqs 9 and 10, ensuring that they kept constant after reaching stationary conditions. This means that every sampling choice was benchmarked with its own optimal set of parameters. The shift was adapted via a second-order scheme^44^ to keep the population close to the target population of 10^5^ for every calculation. The initiator criterion was applied with a population threshold of three.^17−19^ Although we used the initiator approximation in all applications presented here, we would like to emphasize that our new excitation generator applies also to the original (noninitiator) FCIQMC. The data analysis was performed using pyblock, pandas, numpy, and matplotlib.^45−48^ All efficiencies were normalized to the current PCHB algorithm in NECI. Sampling from constrained subsets uses our improved redrawing algorithm (Subsection 3.2) everywhere; for more details, see Appendix A.1.

### 5.2 Efficiency Comparison across Excitation Generators

Table 3 concisely summarizes the efficiency of different combinations of sampling schemes. We refer to the following subsections for a detailed discussion on the results for each test case.

**Table 3:** Efficiency of Different Combinations of Sampling Schemes from Tables 1 and 2 for All Systems^a^

excitation generation		efficiency ratio				
singles	doubles					
*I*:*A*	*I*−*J*:*A*−*B*	H_30_	5·(C_6_H_6_)	10·(C_6_H_6_)	Fe-Por	N_2_
unif:unif	unif-unif:fast-fast^b^	1	1	1	1	**1**
unif:unif	unif-unif:fast-fast	1.00(5)	2.32(9)	2.5(3)	0.883(7)	0.70(1)
unif:unif	full-full:fast-fast^c^	0.99(3)	2.05(8)	2.3(3)	0.92(2)	0.70(2)
unif:fast	unif-unif:fast-fast	**2.9(2)**	1.27(5)	1.2(2)	1.01(5)	0.98(3)
unif:fast	unif-fast:fast-fast	2.8(2)	2.08(7)	1.9(2)	0.354(6)	0.123(4)
unif:fast	full-full:fast-fast	2.7(1)	**2.7(1)**	2.8(4)	0.87(4)	0.79(3)
unif:full	unif-full:fast-fast	2.2(1)	2.5(1)	3.0(4)	0.87(1)	0.70(2)
unif:full	unif-full:full-full	2.1(1)	**2.7(1)**	**3.7(5)**	**1.21(5)**	0.46(2)
unif:full	full-full:fast-fast	2.2(1)	2.22(9)	2.6(3)	0.82(1)	0.78(3)
full:full	full-full:fast-fast	2.0(1)	2.12(5)	2.5(3)	0.77(3)	0.74(3)
full:full	full-full:full-full	1.95(7)	2.34(6)	3.2(5)	1.17(4)	0.45(4)

a The efficiency is given by  and normalized to the currently used algorithm in NECI, which is unif:unifunif-unif:fast-fast. The fastest speedup factor per system is printed bold. The algorithms are lexicographically sorted by sampling quality unif < fast < full. The error for the last digit is given in parentheses.

b The choice in NECI.^3^

c The choice by Holmes et al.^1^

Uniform, fast-weighted, and fully weighted sampling schemes for particles and holes are labeled as unif, fast, and full, respectively. The colon symbol (:) separates particle- from hole-selection. For example, the sampling scheme for a double excitation, *p*_2_^uni^(*I*)|_*I*∈|*D*_*i*_⟩_·*p*_2_^PCHB^(*J*|*I*)|_*J*∈|*D*_*i*_⟩_·*p*_2_^PCHB^(*A*|*IJ*)·*p*_2_^PCHB^(*B*|*IJA*), would be written as unif-full:fast-fast.

In principle, there are 3^6^ = 729 different combinations of samplers, but we restrict ourselves to the handful listed in Table 3, which represent the most meaningful. The conditional probabilities for the holes and for the second particle can use more information as compared to the first particle; hence, the later particle and hole selections should use less uniform sampling. It follows that a full:unif single excitation generator is expected to be less efficient than unif:full, due to the unnecessary weighting of the first particle, which, as shown earlier, in general exhibits uniform distributions.

#### 5.2.1 H_30_-Chain

The first example is a neutral chain of 30 hydrogen atoms that are equally spaced 4.2 *a*_0_ apart. The basis used was STO-3G, which contains a minimal basis set of 30 basis functions, implying that (30,30) is the full-CI space. A *C*_1_ point-group was utilized. Closed-shell Hartree–Fock-optimized orbitals were transformed by a Pipek–Mezey localization^49^ to obtain orthonormal and localized MOs.

Because of the fully atom-localized MOs, the system is dominated by single excitations. If for example, all excitations are selected in a fully weighted manner, then *p*_1_ is adjusted to 98% by eq 10, i.e., mostly single excitations are attempted. This means that the choice of the double excitation generator does not have a large influence on the overall efficiency. In Table 3, we indeed see that the efficiencies are grouped by the excitation generator for the single excitations. The purely uniform generation for single excitations unif:unif performs the worst, since it samples far away holes *A* with the same probability as close ones. The fully weighted excitation generation full:full performs the best per iteration, but it is outperformed by unif:full when taking wall-clock time into account since the choice of the first particle is near-uniformly distributed (see Section 4). The CAS (30,30) has no excess of empty orbitals, and surprisingly, unif:fast is the fastest combination. The fast-weighted hole-selection unif:fast performs better (by roughly  ≈ 30%) than the fully weighted hole-selection unif:full.

This can be explained as follows: due to hermiticity (compare eq 22), we have *S*_*I*_^*A*^ = *S*_*A*_^*I*^. If an excitation *A* ← *I* has a high weight, then the reverse excitation *I* ← *A* has a high weight, as well. If *I* gets only occupied via the excitation from *A*, then *A* is unoccupied when *I* is occupied. Since the single excitations are in good approximation the only excitations, there is no other source that would spawn particles to *I* except the few nearest neighbors apart from *A*. Hence the probability of *A* being unoccupied when *I* is occupied is not independent of the precomputed weights but rather correlated, *p*(*A* ∉ |*D*_*i*_⟩|*I* ∈ |*D*_*i*_⟩) ∝ *p*_1_^PCHB^(*A*|*I*). Numerically, we confirm that if we sample via *p*_1_^PCHB^(*A*|*I*), the rate of valid excitations is around 75%. If we would draw the hole *A* uniformly without guaranteeing the orbital to be unoccupied [i.e., *p*_1_^uni^(*A*|*I*)], the rate of valid excitations would be  ≈ 50%.

The precomputed weighted single excitations improve the performance by a factor of nearly three compared to unif:unif. Since the double excitations do not play a large role in this system, the NECI and Holmes et al. approaches, differing exclusively in the double excitations, exhibit a very similar efficiency.^3,1^

#### 5.2.2 Stack of Benzene Molecules

**Figure 7:**
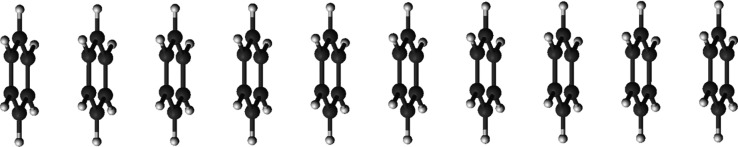
Stack of 10 benzene molecules at a distance of 3.0 Å.

The next example is two stacks of benzene at a distance of 3.0 Å with 5 and 10 molecules, respectively. The stack of 10 molecules is displayed in Figure 7. In both cases, the orbitals were fragment-localized, i.e., natural, Hückel-like orbitals on each benzene. The active space contained all the π and π* orbitals, i.e., a CAS (30,30) for 5 benzene molecules and a CAS(60,60) for 10 benzene molecules. As we have shown in a previous publication,^10^ the system as a whole is dominated by excitations inside each fragment. Additionally incorporating single excitations between the fragments is enough to recover the full CI energy.

In the case of the five molecules, we used the CASSCF (30,30) optimized orbitals from our previous work.^10^ The MOs for the stack of 10 fragments were generated by repeating the MO coefficients of the five fragments along the diagonal to form a block-diagonal coefficient matrix of two blocks. MOs inside each block were still orthogonal to each other, but there was a small overlap between MOs of different blocks; hence, a Gram–Schmidt orthonormalization was applied. This implies that CAS (30,30) is at the energetic minimum with respect to orbital rotations, while CAS (60,60) is only close to it. Hence, the relevance of interfragment single excitations, recovering orbital rotations, is enhanced for the CAS (60,60). In the first case, we have *p*_1_ = 22%, while in the second case, *p*_1_ = 33%, when sampling everything fully weighted.

In Table 3, the efficiencies for different sampling schemes are listed for both stacks of benzene. As expected from the discussion in Sections 3 and 4, we see that uniform selection of the first particle and subsequent fully weighted sampling, i.e., unif:fullunif–full:full–full, works best for both types of systems. It is 3.7 times faster than the current choice in NECI and 60% faster than the choice by Holmes et al. The advantage of not sampling uniformly increases with system size; in fact, the number of direct neighbors stays the same, while the number of distant orbitals increases. We also see that unlike the H_30_ chain, it is important to improve the sampling of both single and double excitations. For example, varying the sampling of doubles by going from unif:fullfull–full:fast–fast to unif:fullunif–full:full–full improves the performance from 2.6 to 3.7.

For these active spaces with a similar number of particles and empty orbitals, the fully weighted hole-selection outperforms the fast-weighted hole-selection, but the difference is perhaps less pronounced than expected. Similar to the H_30_-chain, this can be explained as follows: again, due to hermiticity, we have *W*_*IJ*_^*AB*^ = *W*_*AB*_^*IJ*^ (see eq 12). If excitation *AB* ← *IJ* has a high weight, then reverse excitation *IJ* ← *AB* has a high weight as well. Hence, the probability of *A* and *B* being unoccupied when *I* and *J* are occupied is not independent from the precomputed weights, but rather correlated, i.e., *p*(*A* ∉ |*D*_*i*_⟩|*I*,*J* ∈ |*D*_*i*_⟩) ∝ *p*_2_^PCHB^(*A*|*IJ*). We can confirm this correlation numerically. The hypothetical, uncorrelated rate of a valid hole selection for this active space would be roughly 1/2·1/2 = 1/4, since half of the orbitals are valid picks for the first hole and (half − 1) of the orbitals would be valid picks for the second hole. The numerically obtained rate of valid excitations for fast-weighted hole selection is roughly 50%.

Contrary to the H_30_ chain, the fully weighted hole-selection is still more efficient than the fast-weighted one since there are other effects that lower the aforementioned correlation between PCHB weights and occupation. There are both single and double excitations, and there can be double excitations of the type *IJ* ← *AR*. Hence, it is more probable that excitations happen to *I* or *J* that do not empty *A* and *B* simultaneously.

#### 5.2.3 Fe(II)-Porphyrin

The next system is a Fe(II)-porphyrin model complex whose properties were intensely discussed in our previous publications.^4,5,9,10,50,51^ We use the converged stochastic CASSCF natural orbitals from our previous work^9^ with a (32,34) active space that consists of nine doubly occupied π-orbitals, seven empty π*-orbitals, five metal-centered 3d-orbitals, four doubly occupied σ_N_-orbitals, four empty orbitals of the (4s4p) shell, and five empty *d*′-orbitals (double-shell orbitals). The natural MOs for this system are delocalized but not over the whole molecule. They are still confined to certain regions and can be identified as metal-centered, aromatic, or σ-donating nitrogen orbitals. So there is spatial separation of MOs, but not as strongly as that for the stack of 5 or 10 benzene molecules.

In Table 3, the efficiencies of different sampling schemes are listed for the Fe(II)-porphyrin molecule. As for the stack of benzene molecules, the fastest choice is again unif:fullunif–full:full–full, but the maximum speedup by a factor of 1.21 is much smaller than for the localized benzene stack.

#### 5.2.4 N_2_-Dimer

The last example is full CI on the N_2_ dimer at an equilibrium distance with a Dunning’s cc-pVQZ basis^36^ using Hartree–Fock orbitals, resulting in a (14,110) active space. This system is highly delocalized and has an excess of empty orbitals. The excess of empty orbitals means that a randomly picked index is likely unoccupied, e.g., (*A*|*IJ*)| ≈ *p*_2_^PCHB^ (*A*|*IJ*)|_*A*∉|*D*_*i*_⟩_, which means that the fast-weighted hole-selection outperforms the fully weighted one. On the other hand, it is very important to guarantee orbitals to be occupied in the particle-selection for both single and double excitations. This means that only uniform or fully weighted particle-selection are expected to be performant and explains why unif–fast particle selection for double excitations performs so badly for this system (Table 3).

In the case of single excitations, the approximation of contracting over all orbitals when calculating the weights (eq 22) is particularly poor for these Hartree–Fock orbitals, which means that PCHB weighting for single excitations does not have the same benefits as those for the other systems. For this reason, we see that purely uniform sampling (unif:unif) and uniform sampling of particles followed by fast-weighted selection of holes (unif:fast) show a very similar performance.

In the case of double excitation, there is no approximation regarding the matrix elements that would break down because of the use of Hartree–Fock orbitals. This means that the PCHB weights for doubles are close to the correct weights, and from the discussion so far, we conclude that fast-weighted hole-selection is the fastest method, which we can confirm in Table 3. The probabilities for the first particle *I* are near-uniformly distributed (compare the discussion around Figure 4); hence, the fastest double particle-selection is either unif–unif or unif–full. The first one is faster per wall-clock time, and the latter samples with higher quality. Looking at Table 3, we see that purely uniform particle selection (unif–unif) outperforms unif–full, which makes unif:unifunif–unif:fast–fast the fastest method for this system.

## 6 PCHB and GAS

In the following, we show how, in the stochastic variant of the generalized active space approach (stochastic-GAS), occupation number constraints can be incorporated into the new weighted sampling scheme for singles and how the weighted particle-selection for single and double excitations can improve the GAS performance. In the following, we offer a brief summary of the GAS concept. Details on the stochastic-GAS algorithm can be found in ref (10).

**Figure 8:**
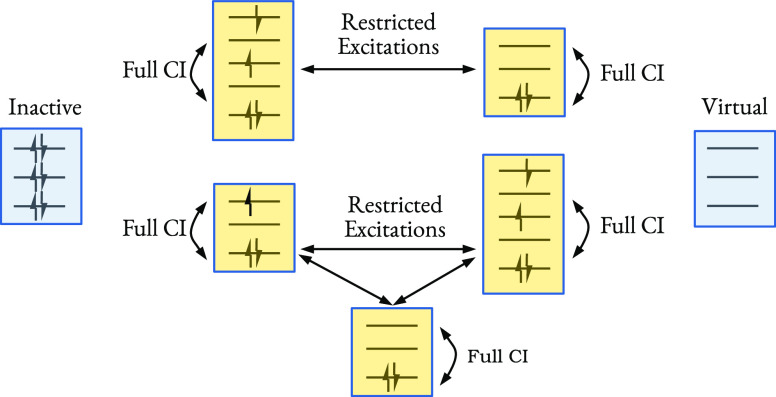
Example of a GASCI wave function.

In GAS, the active orbitals are partitioned into a number of active subspaces. Then only some distributions of particles among these subspaces are allowed, for example, by defining a minimum and maximum particle number per GAS space. A given allowed distribution of particles among the subspaces is called a supergroup.^10,13,52^ For a given supergroup, all possible configurations inside each subspace are allowed, i.e., a full CI wave function inside each subspace is generated. If there is only one supergroup, the GAS spaces are disconnected and no excitations between them are allowed. In Figure 8, a possible GAS wave function is displayed. The GAS algorithm is a natural generalization of other truncated FCI schemes, and it can easily be used to construct restricted active spaces (RAS) (three subspaces) or complete active spaces (CAS) wave function expansions.^13,53−56^

As we showed in our previous work,^10^ the GAS constraints can be included into the PCHB probability distributions with negligible additional run-time cost. The supergroup of a given determinant can be determined by counting the particles per GAS space [a  operation]. Counting how many particles an excitation transfers between GAS spaces is a trivial operation. Hence, for a given supergroup (and all determinants belonging to it), an excitation is GAS allowed if the resulting particle distribution is still inside the chosen GAS constraints. This implies in particular that the starting determinant |*D*_*i*_⟩ is not necessary; only its supergroup is required to determine if an excitation is GAS allowed.

For every supergroup, we can then define modified PCHB probability tables that automatically incorporate the GAS constraints. If we define *i*_sg_ to be a labeling index for the supergroups, we can introduce modified PCHB weights, based on eqs 22 and 12, that are parametrized by the supergroup
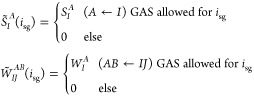
25

At run time, we have to determine the supergroup index for a given determinant (the details of how to do this efficiently are in our previous work^10^) and then draw from the corresponding probability distributions that were precomputed from eq 25.

Apart from the user-defined GAS constraints, there is, due to the Pauli-principle, a “natural” maximum particle number per GAS space, namely, the number of spin–orbitals. The weighted particle selection based on the GAS PCHB weights (eq 25) can use that information and improve the sampling performance.

To see this, let us consider the Anderson impurity model, which consists of one impurity orbital and *n*_bath_ bath orbitals.^57^ The model allows only single excitations and only between the impurity and the bath orbitals, i.e., there may not be any excitations between bath orbitals. If we start from a determinant, where the impurity is doubly occupied, and select a particle on a bath orbital from which to excite, there will be no valid single excitation possible. Since there is a uniform probability of  to select a particle from the bath orbitals, a uniform particle selection will produce invalid excitations with a probability of at least  for such determinants.

This inefficient sampling can be cured with GAS and weighted particle-selection. If we define the impurity orbital to be its own GAS space and the bath orbitals to be in a second GAS space, then there are three allowed supergroups: [2, *N*_e_ – 2], [1, *N*_e_ – 1], and [0, *N*_e_], where we just listed the number of particles per GAS space. If we start now from the supergroup [2, *N*_e_ – 2] and assume that *K* is a spin–orbital index in the bath-orbitals, then we have  for all possible holes *A*. This implies that *p*_1_^PCHB^(*K*|*i*_sg_) = 0 and only one of the two particles in the impurity is selected to be excited, each one with a probability of .

## 7 PCHB and Spin Purification

In this section, we want to show how the modified Hamiltonian
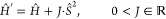
26is automatically efficiently sampled via PCHB. We showed in a previous work how this first-order spin penalty is useful, particularly in FCIQMC, to calculate spin-pure states in a SD basis.^11^

Similarly to the matrix elements of the Hamiltonian (eqs 5–7), we write the matrix elements for the *Ŝ*^2^ operator as
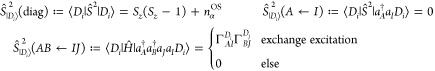
27where *S*_*z*_ is the spin-projection in the *z*-direction, *n*_α_^OS^ is the number of open-shell α electrons of a given determinant, and exchange excitations are all excitations of the type *i*_α_*j*_β_ ← *i*_β_*j*_α_. The diagonal term does not appear in the excitation generation, and the single excitations are not modified at all. Only the double excitations of exchange type are actually modified and their respective PCHB weight (eq 12) becomes |*g*_*AIBJ*_ – *g*_*AJBI*_ + *J*|. If we additionally assume spatial orbitals, i.e., *g*_*AIBJ*_ = *g*_*a*_σ_,*i*_τ_,*b*_μ_,*j*_ν__ = *g*_*a,i,b,j*_δ^σ,τ^δ^μ,ν^, we finally get |*J* – *g*_*ijji*_|.

For a given spin-projection σ, we write the primed σ′ for the opposite spin-projection. We conclude that the particle selection *p*_2_^PCHB^(*j*_σ_|*i*_σ_′) with the modified weight becomes proportional to the spin penalty, i.e., opposite spin pairs are more likely to be picked. The increased probability of exchange interactions depends continuously on *J* and still incorporates the value of the integrals, e.g., *g*_*ijji*_. The same applies to the subsequent hole-selections, which are more likely to sample exchange excitations. Hence, the modified Hamiltonian is efficiently sampled, with a continuous dependence on *J*.

## 8 Conclusions

In this work, we have introduced an improved PCHB algorithm in FCIQMC that takes advantage of the locality of electron correlation and that allows FCIQMC dynamics that are two to four times more efficient than currently available algorithms.

A weighted sampling method for single excitations has been presented that uses precalculated probabilities. The weights for single excitations are obtained by contracting over all orbitals in the two-electron term rather than over only the occupied ones. This approximation works particularly well for localized orbitals, where it can take advantage of the exponential decay of integrals with increasing spatial distance. Precisely in the localized basis, single excitations are pivotal for efficient FCIQMC dynamics; hence, right when weighted sampling of single excitations becomes relevant, our approximation works well.

Also, optimal combinations of uniform, fully weighted, or fast-weighted sampling of particles or holes in double excitations have been identified for a variety of chemical systems, active spaces, and forms of the orbital basis (localized or delocalized). We conclude the following qualitative rules: (a) for localized orbitals and active spaces featuring a similar number of particles and spatial orbitals, the unif:fullunif-full:full-full is generally the fastest combination of sampling schemes, as shown by the fragment-localized benzene stacks and the Fe-porphyrin examples. The speedup was up to a factor of four compared to the current implementation in NECI. (b) If the system uses delocalized orbitals with an excess of empty ones, such as the N_2_ dimer in a large basis, then unif:unifunif-unif:fast-fast is preferred. This combination gives a 50% speedup compared to the choice of Holmes et al.

We have also suggested an alternative method to draw from constrained subsets of precalculated probabilities that is 5–10 times faster than the sampling methods currently available and scales practically constant when keeping the particle number constant and increasing the basis set size. The details are discussed in the Appendix. This improvement can be used in all implementations of PCHB, for both singles and doubles, and improves the performance of fully weighted sampling.

Our new precomputed weighting scheme for single excitations can already be applied in combination with stochastic-GAS and the spin purification approach in FCIQMC, making both methods more efficient.

As a final remark, we would like to highlight that the full strength of the novel precomputed weighting scheme emerges when combined with the spin-adapted implementation of FCIQMC,^8,34^ based on the graphical unitary group approach (GUGA). This will be the subject of a future publication. In a series of recent works,^6−8,12,28−30^ we have shown that by simple orbital localizations and reorderings, it is possible to obtain CI Hamiltonian matrices with a unique and unprecedented block diagonal structure. As a consequence, extremely compact wave functions (to the limit of near-single-reference) are obtained for ground and excited states of exchange-coupled polynuclear transition metal clusters. Since localized bases are propaedeutic to the GUGA wave function compression, it is clear that an excitation generator that exploits locality would further facilitate the screening of unnecessary excitation attempts in such sparse Hamiltonians. Our nonuniform drawing scheme in GUGA would promote those single and double excitations among nearest-neighbors and would screen out excitations that are related to decaying integral values, yielding efficiently sampled excitations in extremely sparse Hamiltonians.

## Figures and Tables

**Figure 9:**
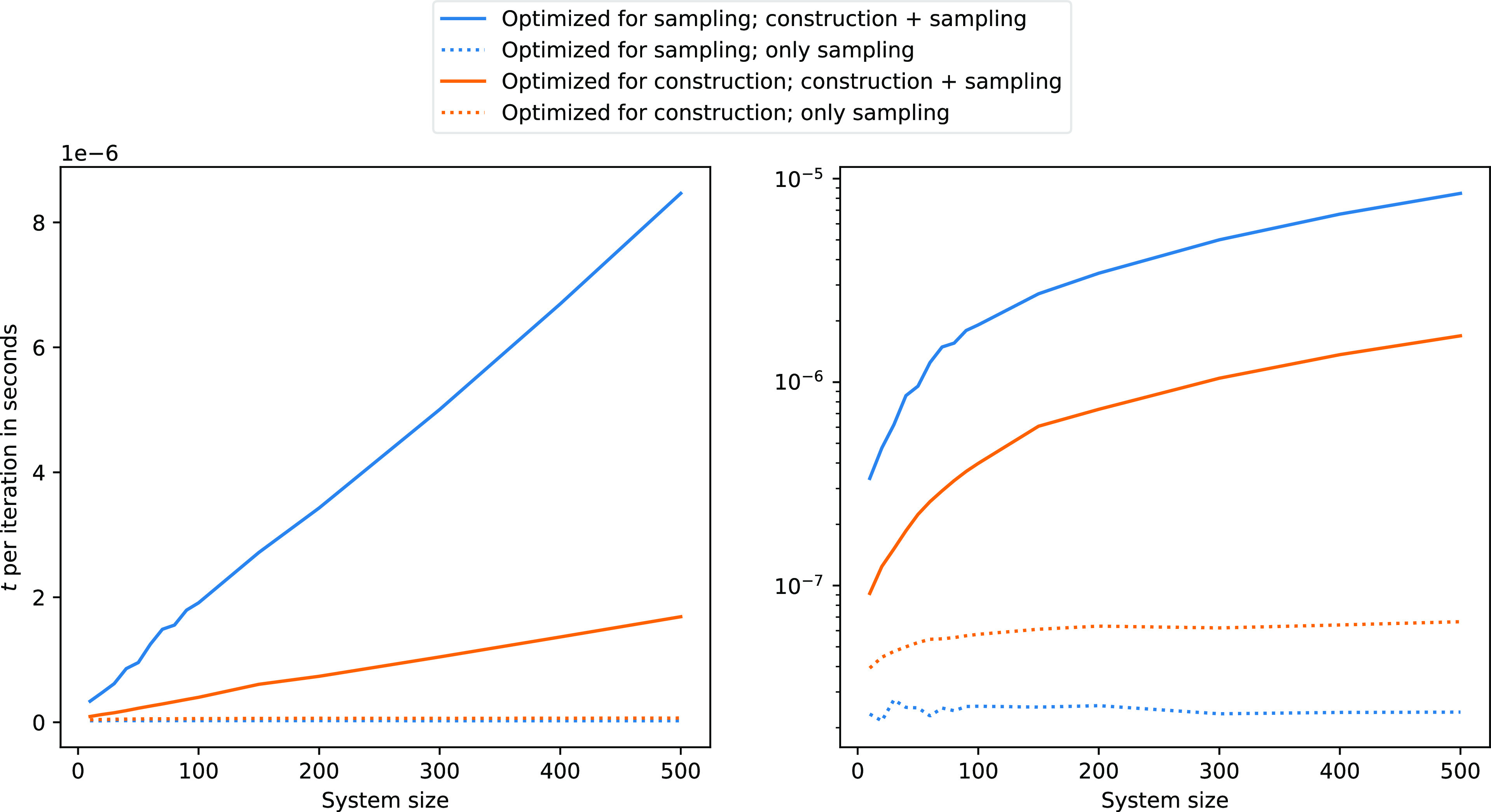
Sampling times of the alias method with and without the optional sorting step in the initialization (compare algorithm 2) against the system size *n*. The dotted lines are purely the sampling times, while the solid lines include the sampling and construction times.

**Figure 10:**
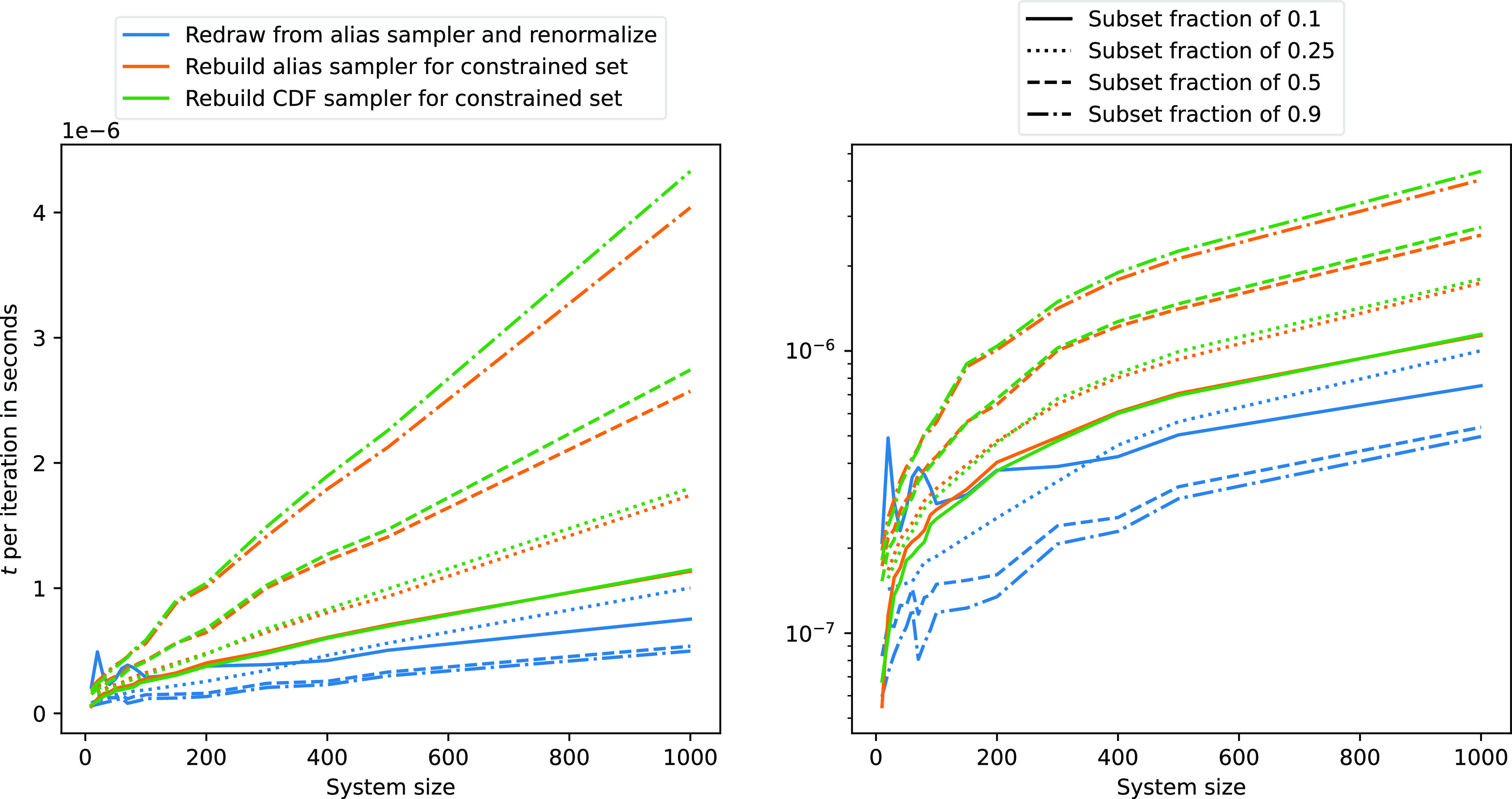
Sampling times for drawing from a constrained subset. The colors denote the algorithm: blue uses redrawing (algorithm 3) orange and green rebuild an alias and CDF sampler for the constrained subset. The line style denotes the size of the subset measured as a fraction of the number of elements.

## Appendix

### A.1 Constrained Sampling

In this section, we will discuss the various ways of how to efficiently sample from constrained subsets of known discrete probability distributions. To do so, we need to recapitulate the two main algorithms for sampling non-uniform, discrete distributions in the FCIQMC community, namely, the cumulative distribution function (CDF) and the alias sampling^23,35^ methods.

The CDF sampling is shown in algorithm 1. It has linear scaling for the initialization and logarithmic scaling for the sampling step.

The alias sampling is shown in algorithm 2. The (cumulative) summation over weights *w*_*i*_ is the same for both methods, but there is additional work in the initialization step of the alias sampling. This means that the scaling in the initialization is at least linear for both methods, but CDF sampling has a smaller prefactor. In the final step of the alias sampling (algorithm 2), there are two if–else branches. Both branches have constant scaling, but the one that returns *K*_⌊*r*⌋_ has an additional indirection. To avoid this case, the sum of *U*_*i*_ has to be maximized in the initialization, which is an NP-hard problem but can be approximately achieved by pairing large *U*_*i*_ with small *U*_*i*_.^58^ Adding this optional sorting step lets the initialization of the alias tables scale with , but the sampling becomes faster.

In addition, there is another trade-off between fast construction and fast sampling for both methods. Often the calling code, in particular FCIQMC, does not want to only sample a random value but wants to also know its probability *p*_*i*_. The necessary renormalization of the weights  does not have to be done at initialization; it could be computed upon the request of the calling code.

To illustrate these effects, we will first compare two implementations of the alias method. The STL std::discrete_distribution implementation of GCC 7.5.0 is optimized for construction, while our own implementation is optimized for sampling time. The C++ code that was used to generate the data is available in the Supporting Information. The code ran on one core of an AMD EPYC 7763 CPU with 2.0 GHz.

In Figure 9, we see that there is a trade-off between optimizing for construction or sampling time. For this reason, we now assume that we optimize for sampling when the sampler is initialized once and then often sampled, while we optimize for construction, if the alias sampler should be constructed often.

Now we can turn our eyes toward an efficient algorithm for sampling from a constrained subset of an already constructed alias sampler. The main idea (algorithm 3) is to just sample via existing alias tables until the sampled index is contained in the subset. Since the alias sampling is so fast, this is usually faster than rebuilding alias or CDF tables for the constrained subsets.

There are some crucial details to improve the scaling, to correctly deal with floating point arithmetic, and to correctly work with zero probability distributions. If we are assuming that the index *i* is contained in the constrained subset *M*, it is drawn with a probability  from it. The renormalization scales linearly with the number of elements in *M*. If *M* contains many elements, it is advantageous to scale with the complement via

28

There are certain situations when all weights and all *p*_*i*_ are zero; then the application of eq 28 would wrongly yield ∑_*j*∈*M*_*p*_*j*_ = 1, although it should be zero. This has to be distinguished from the case where it is indeed ∑_*j*∈*M*_*p*_*j*_ = 1. For this reason, we have to directly evaluate ∑_*j*∈*M*_*p*_*j*_, if the right-hand side of eq 28 is exactly one. There is also another reason that necessitates the direct evaluation. Equation 28 is not exactly true for floating numbers. Since the left-hand side appears in the denominator for renormalization (compare algorithm 3), these small differences can “blow up”, if the overall expression is small. For this reason, the direct calculation is preferred, if *N*_renorm_ ≈ 0. Both cases are caught in line 3.6.

If *N*_renorm_ is nonzero but small, then algorithm 3 will perform many iterations in the while-loop (line 3.14). In this case, it is advised to switch back to CDF sampling (see line 3.10).

It is advantageous to represent *M* as both a sorted enumeration of the contained indices and as a bit mask, since both have an advantage for certain operations. The bit mask allows a very fast lookup to determine *i* ∈ *M* and the complement is just the bit wise NOT. The sorted enumeration can be faster to perform restricted summations over an index.

In Figure 10, we show the performance of constrained sampling via redrawing (algorithm 3) and compare against sampling via rebuilding the CDF or alias tables. The rebuilt alias tables are optimized for initialization, i.e., there is no sorting step in algorithm 2. We restrict the system size to 500 since we assume that calculations in the near future will not exceed 500 spin-orbitals. The subset size, measured as fraction of the number of elements, is varied with the following values {0.25, 0.5, 0.9}. As theoretically expected, all methods scale linearly with the system size, but there is considerable difference between redrawing and rebuilding. Redrawing is near-independent from the subset fraction since the resampling in the while-loop of algorithm 3 is so fast. Only if the subset weight gets minuscule, it is necessary to switch to a rebuilding algorithm (see line 3.10). The rebuilding algorithms on the other hand depend on the subset fraction; the higher the subset fraction, the slower they become. The difference between CDF and alias sampling is negligible for rebuilding and drawing once. If the subset fraction is 0.5, which is the usual case for balanced active spaces with equal number of spatial orbitals and particles, then redrawing is 5 to 10 times faster than rebuilding.

## Supporting Information Available

Geometries, starting orbitals, OpenMolcas inputs, and NECI inputs for our application test cases and the code to time different (constrained) sampling strategies (ZIP)
The appended PDF describes the input files in more detail (PDF)
