# Supplementary material for: Exploiting Locality in Full Configuration Interaction Quantum Monte Carlo for Fast Excitation Generation
Source: J Chem Theory Comput. 2023 Dec 5;19(24):9118–35. doi: 10.1021/acs.jctc.3c00546 (PMC10753814; doi:10.1021/acs.jctc.3c00546)
Supplement: Supplementary file 2 — ct3c00546_si_002.pdf [file ct3c00546_si_002.pdf]

Supporting Information

Exploiting locality in FCIQMC for fast excitation generation

Oskar Weser[1,*], Ali Alavi[1,2], and Giovanni Li Manni[1,*]

[1]Max-Planck-Institute for Solid State Research, Stuttgart, Germany
[2]Yusuf Hamied Department of Chemistry, University of Cambridge, Lensfield Road, Cambridge CB2 1EW, UK
[*]oskar.weser@gmail.com, g.limanni@fkf.mpg.de

The supporting information is made available free of charge. The appended `ZIP` file `SI.zip` contains the following files:

```
sampling_benchmarks/          # files to reproduce the sampling timings
  alias_lib.h
  alias_timing.cpp
  CMakeLists.txt
  constrained_timing.cpp
  README

applications/                 # input to reproduce the applications results
  05_benzene/
    benzene_3.0_Ang.FciInp    # NECI input
    benzene_3.0_Ang.inp       # Molcas input
    benzene_3.0_Ang.InpOrb    # Molcas input orbitals
    benzene_3.0_Ang.xyz       # Geometry
```

```
10_benzene/
 10_benzene_3.0_Ang.FciInp  # NECI input
 10_benzene_3.0_Ang.inp     # Molcas input
 10_benzene_3.0_Ang.InpOrb  # Molcas input orbitals
 10_benzene_3.0_Ang.xyz     # Geometry

FePor/
 tripl.FciInp               # NECI input
 tripl.inp                  # Molcas input
 tripl.InpOrb               # Molcas input orbitals
 3A2g_simple.xyz            # Geometry

H30/
 H30.FciInp                 # NECI input
 H30.inp                    # Molcas input
 H30.InpOrb                 # Molcas input orbitals
 H30.xyz                    # Geometry

N2/
 N2.FciInp                  # NECI input
 N2.inp                     # Molcas input
 N2.xyz                     # Geometry
```
